# PKC Theta Ablation Improves Healing in a Mouse Model of Muscular Dystrophy

**DOI:** 10.1371/journal.pone.0031515

**Published:** 2012-02-14

**Authors:** Luca Madaro, Andrea Pelle, Carmine Nicoletti, Annunziata Crupi, Valeria Marrocco, Gianluca Bossi, Silvia Soddu, Marina Bouché

**Affiliations:** 1 Unit of Histology, and IIM, Sapienza University, DAHFMO, Rome, Italy; 2 Department of Experimental Oncology, Regina Elena Cancer Institute, Rome, Italy; Stem Cell Research Institute, Belgium

## Abstract

Inflammation is a key pathological characteristic of dystrophic muscle lesion formation, limiting muscle regeneration and resulting in fibrotic and fatty tissue replacement of muscle, which exacerbates the wasting process in dystrophic muscles. Limiting immune response is thus one of the therapeutic options to improve healing, as well as to improve the efficacy of gene- or cell-mediated strategies to restore dystrophin expression. Protein kinase C θ (PKCθ) is a member of the PKCs family highly expressed in both immune cells and skeletal muscle; given its crucial role in adaptive, but also innate, immunity, it is being proposed as a valuable pharmacological target for immune disorders. In our study we asked whether targeting PKCθ could represent a valuable approach to efficiently prevent inflammatory response and disease progression in a mouse model of muscular dystrophy. We generated the bi-genetic mouse model *mdx/*θ^−/−^, where PKCθ expression is lacking in *mdx* mice, the mouse model of Duchenne muscular dystrophy. We found that muscle wasting in *mdx/*θ^−/−^ mice was greatly prevented, while muscle regeneration, maintenance and performance was significantly improved, as compared to *mdx* mice. This phenotype was associated to reduction in inflammatory infiltrate, pro-inflammatory gene expression and pro-fibrotic markers activity, as compared to *mdx* mice. Moreover, BM transplantation experiments demonstrated that the phenotype observed was primarily dependent on lack of PKCθ expression in hematopoietic cells.

These results demonstrate a hitherto unrecognized role of immune-cell intrinsic PKCθ activity in the development of DMD. Although the immune cell population(s) involved remain unidentified, our findings reveal that PKCθ can be proposed as a new pharmacological target to counteract the disease, as well as to improve the efficacy of gene- or cell- therapy approaches.

## Introduction

Duchenne muscular dystrophy (DMD) is one of the most common X-linked lethal diseases, and results from the mutation within the gene encoding dystrophin, a large cytoskeletal protein, whose ablation leads to membrane instability [1]. Therapies based on the restoration of dystrophin expression or the administration of dystrophin^+ve^ stem cells are promising, but still in the preclinical phase [2]–[4]. In this context, one of the barrier to successful gene therapy has been recently identified in cellular immunity [5]. Thus, the monitoring of cellular immune responses should be a priority for any experimental therapy designed to increase the number of dystrophin-positive myofibers in patients with Duchenne's muscular dystrophy. In addition, although mechanical injury and membrane defects are important factors promoting dystrophic pathology, increasing evidences highlight aberrant intracellular signalling cascades that regulate inflammatory and immune processes, as key contributors to the degenerative process [1], [6]. Up-regulated inflammatory gene expression and activated immune cell infiltrates are evident during early disease stages in dystrophic muscle, and the identification of specific targets for anti-inflammatory therapies is one of the ongoing therapeutic options. Indeed, glucocorticoids, which have anti-inflammatory properties, are being used to treat DMD with some success; however, the side effects of these drugs often outweigh their benefit [7], [8]. Numerous other anti-inflammatory therapies have been proposed to improve healing [9]–[13]. In this context, it has been recently shown that rapamycin treatment reduced dystrophic phenotype in *mdx* mice and that this effect was associated to a significant reduction in infiltration of T_eff_ cells in skeletal muscle tissue, while T_reg_ cells were preserved [14]. Indeed, a role of lymphocytes activity in the progression of muscular dystrophy is long known, as antibody- or genetic-mediated lymphocyte depletion improved the disease in *mdx*, however, not all studies had produced definitive results, and the implication of lymphocytes and their subtypes in the disease is still to be clearly defined. Among the possible targets, genetic and pharmacological evidences suggest that protein kinase C (PKC) isotypes have essential functions in promoting both early T-cell activation and sustained T-cell adhesion and are proposed as drug targets in adaptive immunity [15]. Among the isotypes, PKCθ is unique in its ability to translocate to the immunological synapses upon T-cell receptor (TCR) activation, regulating NFκB, AP-1, and NFAT transcriptional activity [16]. PKCθ can also directly associate to chromatin, regulating T-cell-specific inducible gene expression program and microRNAs [17]. It is worth noting that PKCθ has been recently shown to be required for full T_eff_ activation while inhibiting T_reg_-mediated suppression [18]. Indeed, PKCθ is being proposed as a particularly attractive target for developing ways to selectively manipulate T_eff_ cell functions that are relevant to pathogenesis of different diseases, including asthma, rheumatoid arthritis, multiple sclerosis and colitis [15], [19]–[21], without imparting a severe immunosuppression. Interestingly, PKCθ is the PKC isoform predominantly expressed also in skeletal muscle, where it mediates various cellular responses [22]–[27]. Although most of these studies demonstrated that PKCθ is actually required for complete histogenesis, differentiation and homeostasis of skeletal muscle, we asked which would be its prevalent role in a context of chronic inflammation, where immune cells activity is a key determinant as in muscular dystrophy. We here demonstrate that immune-cell intrinsic PKCθ activity plays a crucial role in the progression of muscular dystrophy, and targeting PKCθ can be proposed as a valuable therapeutic strategy for the disease.

## Results

### Lack of PKCθ in mdx mice reduces muscle degeneration and inflammation

**Figure 1:**
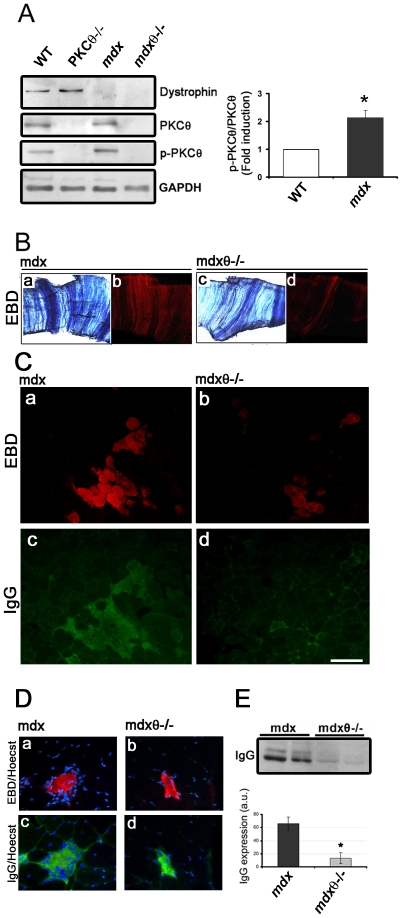
Lack of PKCθ in *mdx* mice reduces muscle degeneration. (**A**) Representative western blot analysis of total protein fraction of TA muscles derived from 2 mo old WT, PKCθ^−/−^, *mdx* and *mdx/θ^−/−^* mice, as indicated. The blot was incubated with the indicated primary antibodies. GAPDH expression level is shown in the bottom for equal loading. PKCθ activation in muscle derived from *mdx* (black bar) mice, expressed as fold induction in respect to WT (white bar, assumed as 1), is shown as the ratio of pPKCθ/PKCθ (right panel), as determined by densitometric analysis from three independent experiments (*p<0.05). (**B**) EBD uptake in diaphragm derived from 2 mo old *mdx* or *mdx/θ^−/−^* mice, as indicated, shown under light (**a, c**) and epifluorescence (**b, d**) microscopy. (**C**) EBD uptake in TA muscle derived from 2 mo old *mdx* (**a**) or *mdx/θ^−/−^* (**b**) mice, as indicated; immunofluorescence analysis of IgG accumulation in *mdx* (**c**) or *mdx/θ^−/−^* (**d**) mice; bar = 200 µm. (**D**) Mononuclear cells accumulation, revealed as Hoechst staining, around single degenerating fiber, detected as EBD uptake (**a–b**) and IgG immunofluorescence (**c–d**), in TA muscles from *mdx* (**a** and **c**) or *mdx/θ^−/−^* (**b** and **d**). (**E**) Representative western Blot analysis of IgG accumulation in TA muscles from *mdx* or *mdx/θ^−/−^* (two mice/genotype), as indicated. Densitometric analysis is shown in the bottom (*mdx*, black bar; *mdx/θ^−/−^*, grey bar, *p<0.05).

**Figure 2:**
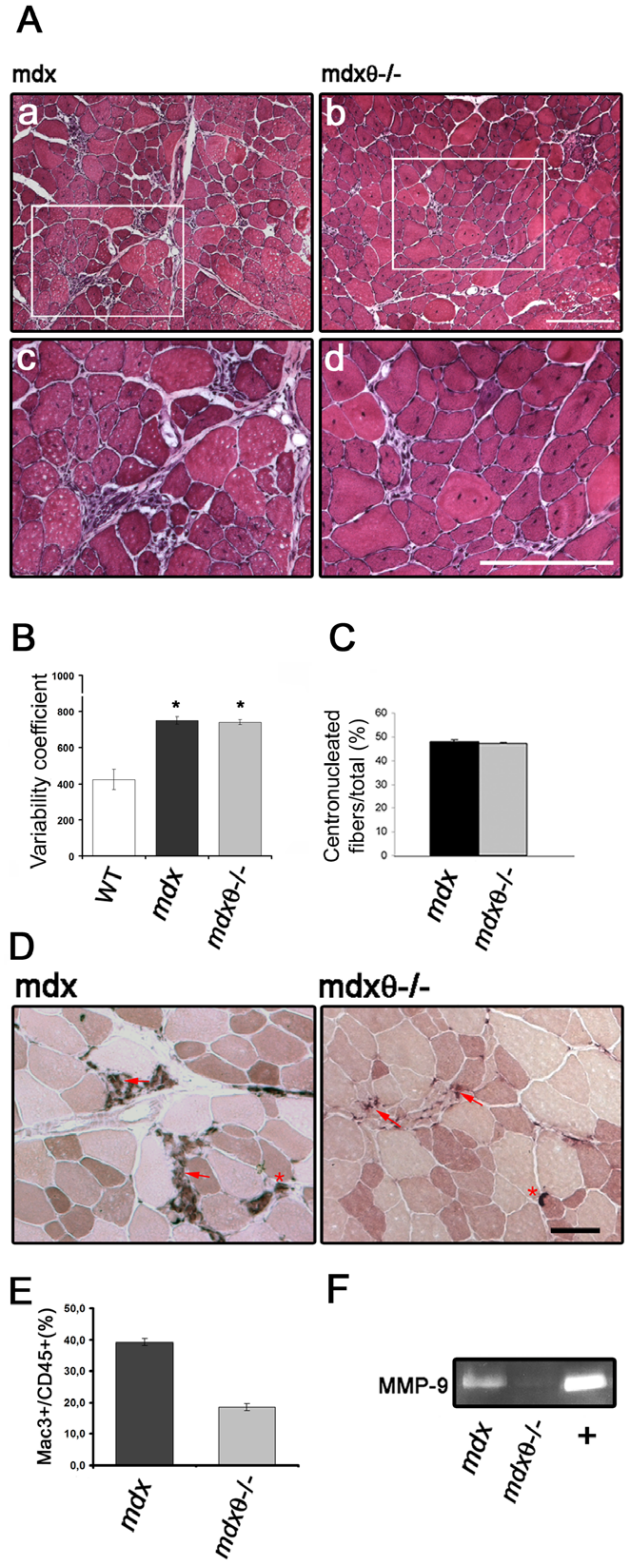
Lack of PKCθ in *mdx* mice reduces cell infiltrate in muscle. (**A**) Hematoxylin/Eosin staining of TA crysosections derived from 2 mo old *mdx* (**a, c**) and *mdx/θ^−/−^* (**b, d**). The insets in **a** and **b** indicate the areas shown in **c** and **d**, respectively, at higher magnification; bar = 100 µm. (**B**) Myofiber variability coefficient in TA muscles derived from 2 mo old WT, *mdx* and *mdx/θ^−/−^*, determined as described in the material and methods sections. (n = 3/genotype). (**C**) Percentage of centrally nucleated myofibers in TA muscles derived from 2 mo old *mdx* and *mdx/θ^−/−^*, expressed as percentage over the total number of myofibers (n = 3/genotype). (**D**) Esterase histochemical staining of TA cryosections derived from 2 mo old *mdx* and *mdx/θ^−/−^* mice, as indicated. Arrows indicate cell infiltrates, arrows indicate neuromuscular junctions. Bar = 200 µm (**E**) FACS analysis of CD45^+ve^/Mac3^+ve^ mononucleated cells isolated from TA muscle derived from 2 mo old *mdx* and *mdx/θ^−/−^* mice, as indicated, expressed as percentage of the total number of cells examined. The percentage of reduction in *mdx/θ^−/−^*muscle, in respect to *mdx*, is also shown (n = 3/genotype). (**F**) Gel zymography of MMP9 activity in TA muscle derived from 2 mo old *mdx* and *mdx/θ^−/−^* mice, as indicated; media collected from differentiating muscle cell cultures was used as positive control (+).

**Figure 3:**
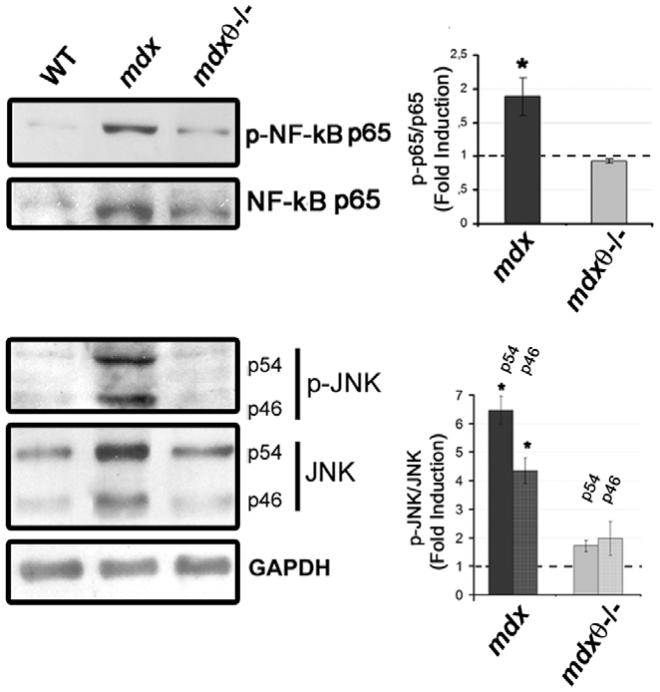
Lack of PKCθ in *mdx* mice prevents up-regulation of pro-inflammatory pathways. Left panel: representative Western Blot analysis of total protein fraction of TA muscles derived from 2 mo old WT, *mdx* and *mdx/θ^−/−^* mice, as indicated. The blot was incubated with the anti- p-NF-kB p65, NF-kB p65, - p-JNK, - JNK antibodies, as indicated. GAPDH level of expression was used for normalization. The p-p65NFkB/p65NFkB (top) and of p-JNK/JNK (both p46 and p54) (bottom) ratio, as determined by densitometric analysis from three independent experiments, is shown in the right, expressed as fold induction in respect to WT (assumed as 1, dotted line). *p<0.01 in respect to WT.

To verify whether inhibition of PKCθ may improve healing in muscular dystrophy, we crossed the PKCθ knock out model (PKCθ^−/−^) with *mdx*, the mouse model of DMD. We first observed, by Western blot analysis, that PKCθ was highly expressed in both WT and *mdx* hindlimb muscle, but a significantly higher portion of it was phosphorylated, as a feature of its activation [16], in the *mdx*, as compared to WT (**Fig. 1A**). As expected, no dystrophin expression was detectable in the *mdx* and *mdx*/θ^−/−^ muscle, and no PKCθ immunoreactivity in the *mdx*/θ^−/−^ muscle (**Fig. 1A**). Both mutants were healthy at birth. We thus evaluated myofiber degeneration in diaphragm (as being diaphragm one of the most affected muscle group in *mdx*) and in tibialis anterior (TA) of 2 mo old mice, as Evan's blue dye (EBD) uptake. As shown in **Figure 1B and C** (**a–b**), myofiber degeneration was significantly reduced in both muscles derived from *mdx/θ^−/−^* in respect to *mdx*. As expected, those degenerating areas were sites of a robust inflammatory response, as shown by anti-mouse IgG immunofluorescence (**Fig. 1C, c–d**). Indeed, while degenerating fibers in *mdx* muscle were surrounded by many mononucleated cells, degenerating fibers in *mdx/θ^−/−^*were almost avoided of surrounding mononucleated cells, and mouse IgGs were strictly localized on the fiber itself (**Fig. 1D**). Accordingly, Western Blot analysis revealed that the IgG content in the protein extract from *mdx/θ^−/−^* muscle was greatly lower than that from *mdx* (**Fig. 1E**). Hematoxilin/Eosin (H/E) staining of TA muscle sections, showed that lack of PKCθ resulted in significant reduction in cell infiltrate as compared to *mdx* (**Fig. 2A**), leading to an overall maintenance of muscle structure; however, the myofibers variability and the percentage of centro-nucleated myofibers over the total number of fibers (as features of dystrophic muscle) were similar between the two genotypes (**Fig. 2B–C**). As being macrophage infiltration the most prominent immune feature observed in *mdx* [6], TA cryosections were analysed for esterase activity. As shown in **Figure 2D**, the areas of macrophages infiltrate in 2 mo old *mdx*/θ^−/−^ muscle were strongly reduced; accordingly, FACS analysis of CD45/Mac-3 co-expressing cells, revealed that the level of macrophages was reduced by ≈50% in 2 mo old *mdx*/θ^−/−^ muscle, as compared to *mdx* (**Fig. 2E**). Macrophages are known to store and produce matrix metalloproteinase 9 (MMP-9) in response to different stimuli, such as oxidative stress from necrotic tissue, and represent the major source of MMP-9 [28]. Indeed, the high level of MMP9 activity observed in *mdx*, was strongly prevented in *mdx*/θ^−/−^ muscle, as shown by zymography (**Fig. 2F**). Moreover, as shown in **Figure 3** **,** lack of PKCθ strongly prevented the hyper-expression/activation of pro-inflammatory signalling pathways; in fact, both the level of expression and of phosphorylation of the p65 subunit of NFkB and of JNK in *mdx/θ^−/−^* TA muscle were much lower than in *mdx*, very similar to the level observed in WT. The p65 subunit of NFkB is the NFkB subunit mostly hyperactive in *mdx* muscle [11] and JNK is the upstream regulator of AP1 signalling pathway, which is also hyperactive in *mdx* [1].

### Lack of PKCθ in mdx mice improves muscle regeneration

**Figure 4:**
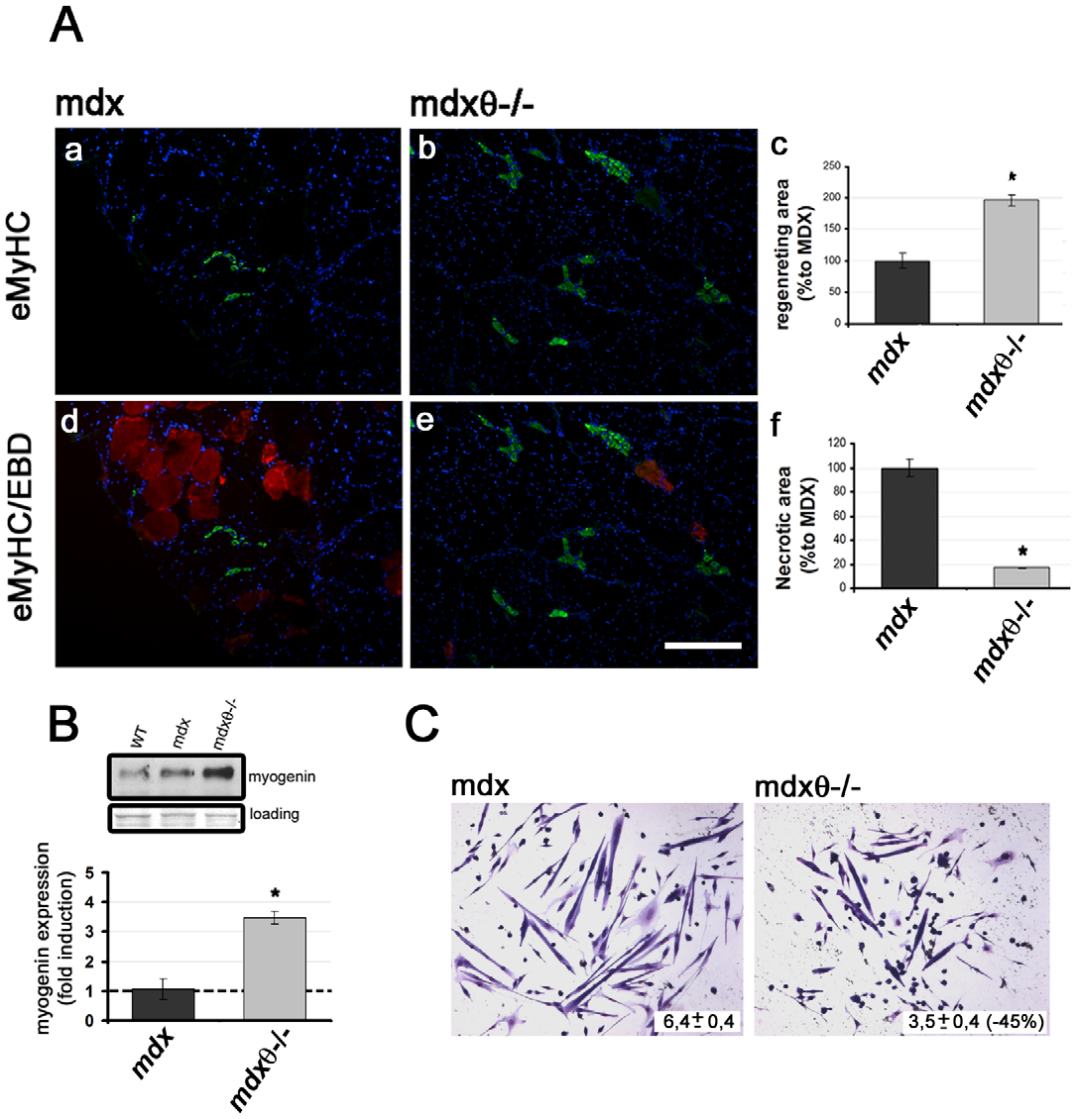
Lack of PKCθ in *mdx* mice improves muscle regeneration. (**A**) eMyHC immunofluorescence (green) in TA cryosections derived from *mdx* (**a** and **d**) and *mdx/θ^−/−^* (**b** and **e**) mice, as indicated. Merge with EBD uptake (red) is shown in **d** (*mdx*) and **e** (.*mdx/θ^−/−^*). Bar = 200 µm. Extension of regenerating, eMyHC^+ve^, area (**c**) and of necrotic, EBD^+ve^, area (**f**) in *mdx/θ^−/−^*, expressed as the percentage in respect to the respective areas in *mdx* (assumed as 1); *p<0.01, n = 3/genotype. Bar = 200 µm. (**B**) Representative Western Blot analysis of total protein fraction of TA muscles derived from 2 mo old WT, *mdx* and *mdx/θ^−/−^* mice, as indicated, incubated with the α-myogenin antibody; Red Ponceau staining of the membrane is shown for equal loading. Up-regulation of myogenin expression in *mdx/θ^−/−^*, in respect to *mdx* (assumed as 1), muscles, as determined by densitometric analysis of three independent experiments is shown in the bottom (n = 3/genotype) (**C**) Representative Wright staining of *mdx*- and *mdx/θ^−/−^*- muscle derived cells, as indicated, cultured in DM for 48 hrs. The mean number of nuclei contained within each myotube is shown, as well as the percentage of reduction in *mdx/θ^−/−^* in respect to *mdx*, as determined from three independent experiments.

To verify whether the observed reduction in muscle wasting and cell infiltrate came along with improvement in muscle repair, muscle regeneration was analysed in the mutant mice by both morphological and biochemical analyses. Immunofluorescence analysis of embryonic myosin (eMyHC) expression, a marker of regenerating myofibers, revealed that lack of PKCθ resulted in an increase on eMyHC expressing myofibers along with a reduction in EBD positive myofibers (**Fig. 4A**). Indeed, quantitative analysis revealed that the extension of “regenerating area”, including eMyHC expressing fibers, was significantly higher in *mdx*/θ^−/−^, as compared to *mdx* muscle, parallel to a significant decrease in “degenerating area”, including EBD stained fibers (**Fig. 4A**). Accordingly, the level of myogenin expression, a marker of differentiating myoblasts, was strongly increased in *mdx*/θ^−/−^, as compared to *mdx* muscle (**Fig. 4B**). Intriguingly, we previously showed that PKCθ is actually required for myofiber growth both *in vivo* and *in vitro*, as being an upstream regulator of the expression of pro-fusion genes [27]. To unravel the apparent contradiction with our current results, *in vitro* differentiation of primary myoblasts derived from *mdx* and from *mdx*/θ^−/−^ hindlimb muscle was compared. As shown in **Figure 4C**, by 48 hours in DM, *mdx* myoblasts had formed elongated myotubes containing a large number of nuclei; by contrast, *mdx/θ^−/−^* myoblasts formed thinner myotubes with reduced number of myonuclei, according to our previous observation in PKCθ^−/−^ myoblasts. Taken together, these results suggest that lack of PKCθ in *mdx* makes a more favourable environment for muscle precursor cells to differentiate, rather than enhancing their activation/differentiation ability. Indeed, though the regenerating area *in vivo* was more extensive in *mdx* mice lacking PKCθ, as compared to *mdx*, cross-sectional area of individual eMyHC, regenerating myofibers was medially reduced by ≈15%, as a result of PKCθ.ablation.

### Lack of PKCθ in mdx mice preserves exercise performance

**Figure 5:**
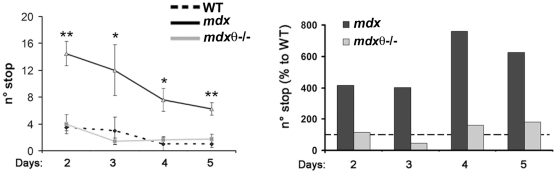
Lack of PKCθ in *mdx* mice preserves exercise performance. Treadmill exercise test performed on 2 mo old WT, *mdx* and *mdx/θ^−/−^* mice (n = 6 each genotype) for a 30 min running, twice a week, for 3 weeks. The average number of stops recorded from mice of each genotype in each day, during the 30 min running, is shown in the left (WT, black line; *mdx*, dotted line; *mdx/θ^−/−^*, grey line). The extreme time points (1st and 6th days) performance were not included. Same results are expressed as percentage in respect to WT mice (assumed as 100%; *mdx/θ^−/−^*, grey bars; *mdx*, black bars) in the right, for each day performance.

To verify whether the improvement in muscle maintenance and regeneration resulted in better performance, a treadmill endurance test was performed and the number of times the mice stopped during the 30 min running, each day of the test, was recorded. As expected, *mdx* mice stopped increasing times during individual running (not shown) and, medially, many more times than WT mice, in each day examined (**Fig. 5**). Importantly, *mdx*/θ^−/−^ mice behaved very similar to WT, both during the running than in all days examined (**Fig. 5**).

### Rescue of PKCθ expression in hematopoietic cells in mdx/θ^−/−^ mice, by bone marrow transplantation, partly restores mdx mice phenotype

**Figure 6:**
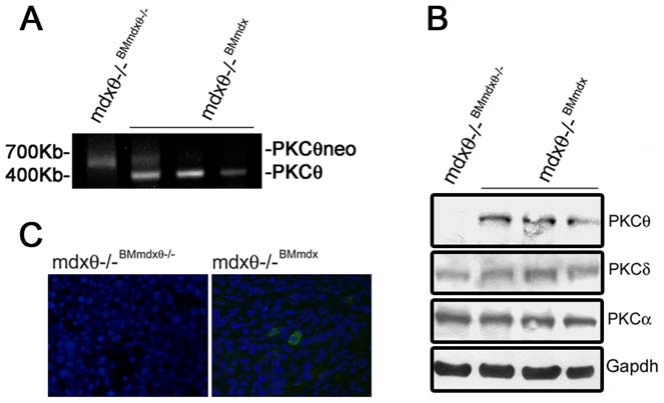
Rescue of PKCθ expression in hematopoietic cells in *mdx/θ^−/−^* mice, by bone marrow transplantation. (**A**) PCR analysis of BM cell suspension genomic DNA derived from *mdx/θ^−/−BMmdx/θ−/−^* and from *mdx/θ^−/−BMmdx^*, as indicated. The reappearance of the 400 kb PCR product from the PKCθ gene in *mdx/θ^−/−BMmdx^* is shown. (**B**) Western Blot analysis of PKCθ, PKCδ and PKCα expression in total protein extract from thymi derived from *mdx/θ^−/−BM mdx/θ−/−^* and from *mdx/θ^−/−BMmdx^*. (**C**) Immunofluorescence analysis of PKCθ-expressing cells (green) in the spleens derived from *mdx/θ^−/−BMmdx/θ−/−^* and from *mdx/θ^−/−BMmdx^*. Hoechst was used to counterstain the nuclei (blue).

**Figure 7:**
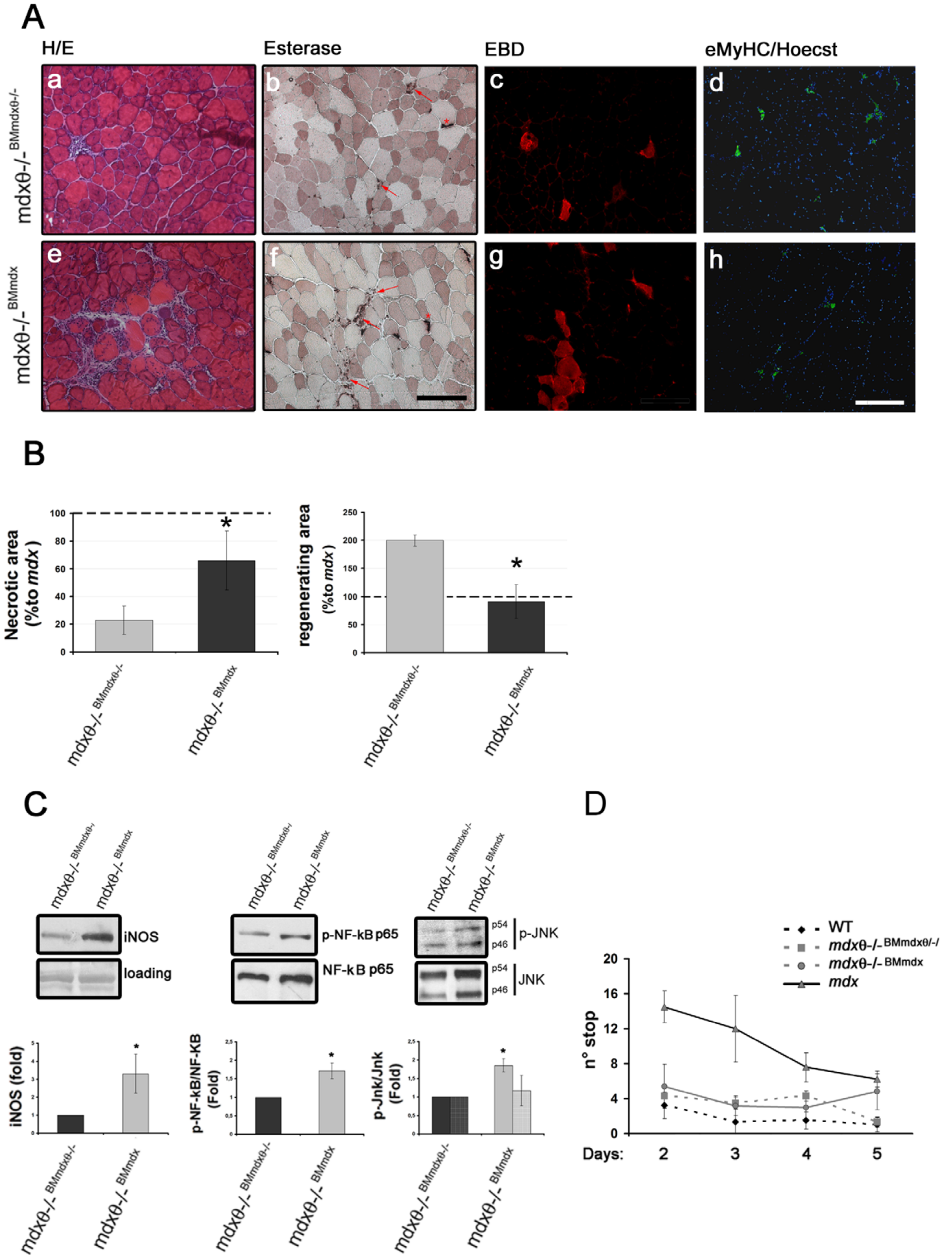
Rescue of PKCθ expression in hematopoietic cells in *mdx/θ^−/−^* mice restores *mdx* mice phenotype. (**A**) H/E (**a** and **e**) and esterase (**b** and **f**, arrows indicate cell infiltrates, asterisks indicate neuromuscular junctions) as well as EBD uptake (**c** and **g**) and eMyHC immunofluorescence (**d** and **h**, Hoechst was used to counterstain the nuclei) of TA cryosections derived from *mdx/θ^−/−BMmdx/θ−/−^* (**a–d**) and from *mdx/θ^−/−BMmdx^* (**e–h**) mice. Bar = 200 µm. (**B**) Extension of regenerating, eMyHC^+ve^, area and of necrotic, EBD^+ve^, area in *mdx/θ^−/−BMmdx/θ−/−^* and in *mdx/θ^−/−BMmdx^* expressed as the percentage in respect to the respective areas in *mdx* (assumed as 1), *p<0.05, n = 3/genotype; (**C**) Western Blot analysis of iNOS expression, and of NFkB and JNK expression and activation (phosphorylation) in protein extracts from TA muscle derived from *mdx/θ^−/−BMmdx/θ−/−^* and from *mdx/θ^−/−BMmdx^*; red Ponceau staining of the membrane is shown (loading) for equal loading. Representative blots are shown. The level of expression or of the activation of the above molecules was determined by densitometric analysis and expressed as fold induction in *mdx/θ^−/−BMmdx^* in respect to *mdx/θ^−/−BMmdx/θ−/−^*, assumed as 1, evaluated from 3 separate experiments (bottom). *p<0.05 (**D**) Treadmill exercise test performed on *mdx/θ^−/−BMmdx/θ−/−^* (squares, dotted grey line) and *mdx/θ^−/−BMmdx^* (circles, grey.line) mice for a 30 min running, twice a week, for 3 weeks, as above, starting 6 weeks after transplantation (n = 3/genotype). Performance of *mdx* (triangles, black line) and *mdx/θ^−/−^* (rhombi, dotted black line) mice is also reported for comparison.

To verify whether the obtained phenotype was actually due to alterations in inflammatory cells activity, 1 mo old *mdx* bone marrow (BM) was transplanted into irradiated, age and sex matching *mdx*/θ^−/−^ mice. In these mice, hereafter called *mdx*/θ^−/−BM*mdx*^, PKCθ was thus expressed only in the BM-derived cells. As control, another group of age and sex matching *mdx*/θ^−/−^ mice was transplanted with BM derived from *mdx*/θ^−/−^,hereafter called *mdx*/θ^−/−BM*mdx*/θ−/−^. The mice were sacrificed 6 weeks after transplantation and BM repopulation was ensured by genomic PCR analysis, as the appearance of the PKCθ WT PCR product (**Fig. 6A**). Western blot analysis revealed that PKCθ expression was largely rescued in thymus derived from *mdx*/θ^−/−BM*mdx*^ transplanted mice, while the expression of other PKC isoforms was unaltered (**Fig. 6B**), and immunofluorescence analysis revealed that PKCθ expressing cells were detectable in the spleen (**Fig. 6C**), demonstrating that engrafted hematopoietic cells repopulated also mature compartments. H/E staining of TA muscle crysections derived from *mdx*/θ^−/−BM*mdx*^ revealed a significant increase of infiltrating cells (**Fig. 7A, e**), as compared to those derived from *mdx*/θ^−/−BM*mdxθ*−/−^ (**Fig. 7A, a**), which were mostly macrophages, as shown by esterase histochemistry (**Fig. 7A, f**). Moreover, EBD uptake revealed an increase in degenerating myofibers in the *mdx*/θ^−/−BM*mdx*^ (**Fig. 7A, g**), as compared to *mdx*/θ^−/−BM*mdxθ*−/−^ (**Fig. 7A, c**), parallel to a reduction in eMyHC expressing fibers (**Fig. 7A, h**) to levels comparable to *mdx*, as shown by quantitative analyses (**Fig. 7B**). Accordingly, iNOS expression, as a marker of macrophage infiltration, increased, as well as both NFkB and JNK expression and activity (**Fig. 7C**). However, the observed worsen phenotype was not translated in worsened exercise performance, as both *mdx*/θ^−/−BM*mdx*^ and *mdx*/θ^−/−BM*mdxθ*−/−^ behaved similar to WT mice in a treadmill endurance test (**Fig. 7D**).

## Discussion

We show in this article that lack of PKCθ in *mdx* prevents muscle wasting while improving muscle structure, regeneration and performance. This phenotype is associated to, and, probably, dependent on, reduced inflammation, which could make a more favourable environment for muscle precursor cells to differentiate.

The *mdx* mouse strain is the most widely used animal model for DMD; although it presents a milder phenotype compared to DMD, the immune cell populations in their muscles resemble those seen in DMD patients [6]. As first, we found that PKCθ is highly phosphorylated/active in *mdx* muscle as compared to WT muscle, and its lack significantly reduced muscle necrosis evaluated as EBD positive fibers in both DIA and TA. Interestingly, not only the percentage of degenerating myofibers was reduced, but also were sites of IgG accumulation. As PKCθ deficient mice have been shown to mount a significantly reduced lung inflammation response to antigen challenge and exhibit reduced inflammation in rodent models of arthritis as well as in autoimmune disease, the possibility that the improved phenotype was dependent on its activity on promoting inflammation is thus reasonable. Indeed, we show that lack of PKCθ in *mdx* resulted in significantly reduced macrophages infiltration, as revealed by both IHC and FACS analysis. Macrophages are the primary immune cells evident in skeletal muscle of *mdx* mice together to T-cells and neutrophils, as being macrophage infiltration the most prominent immune feature observed [6]. The reduction in macrophages is probably responsible for the reduction in the MMP-9 accumulation observed. Matrix metalloproteinases (MMPs), a family of zinc-dependent endopeptidases, have been shown to play an important role in ECM degradation, inflammation, fibrosis, and activation of latent cytokines and cell adhesion molecules in different pathophysiological conditions, including muscular dystrophies [28]. In particular, the expression of MMP-9 is increased in dystrophic muscle and its inhibition, either genetic or pharmacological, considerably reduces inflammatory response, fibrosis, and enhances the myofiber regeneration in *mdx* mice [28]. Moreover, we show that lack of PKCθ prevented the hyper-activation of the pro-inflammatory pathways NFkB and AP1, known to be up-regulated in *mdx*. It is worth noting that chronic activation of NF-kB signalling is required for DMD pathology by acting both on immune cells and damaged skeletal muscles to promote inflammation and to inhibit myogenic differentiation of muscle precursors [29]. Genetic and pharmacological studies demonstrated that inhibition of NFkB signalling reduced inflammation and necrosis enhancing regeneration and implicated the NFkB signalling pathway as a potential therapeutic target for this disease [11], [30]. Also AP-1 activity is increased early in *mdx* muscle, and is supposed to be implicated in the activation of many inflammatory cytokines and chemokines [31]. Thus, the observed reduction in these pathways in *mdx* muscle lacking PKCθ may contribute to favour muscle healing. It is well known that the inflammatory response negatively contributes to the limited ability of dystrophic muscle to regenerate. Indeed, muscle regeneration is the initial response to muscle damage, but, in Duchenne muscular dystrophy, muscle progenitor cells activation/differentiation is limited [32], [33]. This fact depends mainly to the exhaustion of muscle progenitor cells pool, due to the continuous cycles of degeneration/regeneration [32], [33], but also chronic inflammation contributes to make an unfavourable environment for their activation/differentiation. As we show here, lack of PKCθ in *mdx* actually favoured muscle regeneration, as demonstrated by the increase in eMyHC expressing fibers and by myogenin up-regulation. In this context, sustained myogenin expression in *mdx/θ^−/−^* muscle may theoretically contribute to myofiber metabolic changes, which, in turn, may be involved in muscle wasting prevention and physiology improvement. In fact, several observations suggested that myogenin may participate in at least a part of a fast-to-slow fiber-type transition [34], [35]. Although further analyses are required, no alterations in the level of metabolic enzymes were observed in *mdx/θ^−/−^* muscle, as compared to *mdx* (Madaro et al. unpublished observation). The observed myogenin up-regulation should thus primarily reflect the increase in differentiating satellite cells, as a feature of regenerating muscle, rather than a sustained myogenin expression. These results may appear in contrast with our previous observation showing that PKCθ^−/−^ regenerating muscle displayed the characteristics of delayed regeneration, as compared to time-matching regenerating WT muscle. However, those effects were not due to an impairment in muscle precursors activation/differentiation, rather to an impairment of the late phases of growth/regeneration process, delaying further addition of myonuclei to newly formed myofibers, due to reduction in the expression of the so-called pro-fusion genes. Indeed, we show in this paper that primary cells derived from *mdx/θ^−/−^* muscle formed thinner myotubes with reduced number of myonuclei *in vitro*, as we observed in PKCθ^−/−^ muscle cells, when compared to those derived from *mdx* muscle. It is thus conceivable that in *mdx* chronic inflammation contributes significantly in preventing activation of muscle precursor cells, which however are able to efficiently differentiate, as they do *ex vivo*. By contrast, lack of PKCθ, though delaying the fusion process itself, makes a more favourable environment for muscle precursors cells to differentiate by reducing inflammation, thus allowing muscle repair. Indeed, *mdx/θ^−/−^* regenerating myofiber CSA was reduced, as compared to *mdx*. As a result, muscle tissue structure is preserved, though the myofiber CSA variability and the percentage of centrally-nucleated myofibers were similar to those in *mdx*. Importantly, lack of PKCθ preserved exercise performance in *mdx*, suggesting that the improved phenotype correlates to improved functionality of the muscle. Taken together, these results demonstrate that lack of PKCθ in *mdx* mice dramatically reduces cell infiltration and inflammation, improving muscle regeneration and performance, suggesting a hitherto unrecognized crucial role of PKCθ in promoting immune response in muscular dystrophy. Consistent with this hypothesis, PKCθ expressing BM derived cells partly counteracted improvement in inflammation, muscle wasting and regeneration in *mdx/θ^−/−^* mice, as shown by BM transplantation experiments. Intriguingly, exercise performance was not impaired, instead. This fact may depend on the time required for muscle to decline its functionality: it is conceivable that 6 weeks upon BM transplantation were sufficient to partly restore immune response, which however was still less than in age-matching *mdx*, but not to worsen muscle performance. Although the specific cell population(s) involved is not clear yet, an attractive hypothesis would be that lack of PKCθ prevents T_eff_ cells activity, while maintaining T_reg_ cells activity, which in turn may modulate macrophages phenotype and activity. However, as PKCθ lacks in all cells in the *mdx/θ^−/−^* model, the possible contribution of other tissue components, in particular skeletal muscle, in the observed phenotype cannot be ruled out. In any case, the results obtained demonstrate that PKCθ expression/activity in immune cells is required for the robust inflammatory response in *mdx*, which, in turn, exacerbates the muscle pathology. Further studies are needed to verify long term efficacy, as well as the effect of targeting PKCθ in older animals, when pathological features are already established. Moreover, as being muscular dystrophy a multi-factorial disease, in which inflammation plays a crucial role, the possibility to combine PKCθ targeting as anti-inflammatory therapy, to other gene- or cell- based therapeutic approaches to significantly improve and optimize the therapeutic efficacy, should be explored [5]. The availability, and the ongoing development, of specific PKCθ inhibitors, some of which are already in clinical trials for immune disorders, thus opens new perspectives for pharmacological approach to muscular dystrophy [15], [19].

## Methods

### Mice

PKCθ^−/−^ mice were gently provided by Prof Dan Littman [36]. *Mdx* mice were purchased from Charles River. The mice were cross-bread to generate *Mdx/θ^−/−^* double mutant mice. C57BL10 WT mice (Charles River) were used as control. The animals were housed at the Histology Unit accredited animal facility, in individual cages in an environmentally controlled room (23°C, 12-h light-dark cycle) and provided food and water *ad libitum*. All the procedures were approved by the Italian Ministry for Health and conducted according to the US National Institutes of Health guidelines.

### Antibodies

The anti-PKC-θ and the anti-phospho^Thr538^ PKC-θ, the anti-p65NFkB, the anti-phospho p65NFkB, the anti- JNK and the anti-phospho JNK rabbit polyclonal antibodies were purchased from Cell Signalling Inc., Danvers, MA, USA; the anti-iNOS mouse monoclonal antibody was from BD Bioscience, CA, USA, while the anti-dystrophin was from Leica Microsystems, Germany; the anti-myogenin F5D and the anti-embryonic myosin heavy chain F1.652 mouse monoclonal antibodies were from Developmental Studies Hybridoma Bank, Iowa City, Iowa, USA.

### Primary muscle cell cultures

Primary cultures were prepared from total limb muscles of *mdx* or *mdx/θ^−/−.^* mice, as previously described [27]. Muscle derived cells were grown on collagen-coated dishes, in growth medium, GM (Dulbecco's modified Eagle's medium, D-MEM containing 20% HS, 3% EE, all from Gibco Invitrogen, Carlsbad, CA, USA) in a humidified 5% CO_2_ atmosphere at 37°C. Differentiation was induced by replacing the medium with medium containing lower serum and EE concentration, DM (D-MEM containing 5% HS, 0,75% EE). The cells were fixed after 48 hrs in DM and stained with Wright's solution. The mean number of nuclei contained within each myotube was determined as previously described [27]. Approximately 100 myotubes were counted per dish.

### Histological and immunohistochemical analyses

Muscle cryosections were fixed in 4% paraformaldehyde (Sigma-Aldrich, MO, USA) on ice. Myofiber necrosis was evaluated on muscle cryosections prepared from mice intraperitoneally injected with a 1% Evan's Blue Dye solution (EBD, Sigma-Aldrich, MO, USA), at 1% volume relative to body mass, between 16 and 24 h prior to tissue sampling. The samples were analyzed under an epifluorescence Zeiss Axioskop 2 Plus microscope. For histological analysis, muscle cryosections were stained with Hematoxylin/Eosin solution (Sigma-Aldrich, MO, USA). The muscle fiber mean cross sectional area (CSA) was determined by measuring CSA of fibres in the entire section, using Scion Image 4.0.3.2 software (NIH, Bethesda, MA, USA). Myofiber variability was determined by multiplying the standard deviation of all measurements by 1,000 and dividing it by the mean fiber diameter [37]. Esterase localization and activity was evaluated on cryosections by esterase staining (α-naphthyl butyrate/hexazotized pararosaniline).

Immunofluorescence analysis on crysections was performed as previously described [27]. Nuclei were counterstained with Hoechst 33342 (Fluka, WI, USA) and the samples were analyzed under an epifluorescence Zeiss Axioskop 2 Plus microscope.

### Gelatin zymography

Muscle extracts were prepared in non-reducing lysis buffer [50 mM Tris-Cl (pH 8.0), 200 mM NaCl, 50 mM NaF, 0.3% IGPAL CA-630 and protease inhibitors], as previously described [28]. Equal amount of proteins was separated on 8% SDS–PAGE containing 1 mg/ml gelatin B (Fisher Scientific) under non-reducing conditions. Gels were washed in 2.5% Triton X-100 for 1 h at room temperature followed by incubation in reaction buffer [50 mM Tris–HCl (pH 8.0), 50 mM NaCl, 5 mM CaCl2 and 0.02% sodium azide] for 48 h at 37°C. To visualize gelatinolytic bands, gels were stained with Coomassie Brilliant Blue dye, followed by extensive washing in destaining buffer (40% methanol/10% acetic acid).

### Western blot analysis

Tissue samples were homogenized in ice-cold buffer containing 20 mM Tris (pH 7.5), 2 mM EDTA, 2 mM EGTA, 250 mM sucrose, 5 mM DTT, 200 mg/ml leupeptin, 10 mg/ml Trasylol, 1 mM PMSF, and 0.1% Triton X-100, as previously described [27]. An equal amount of protein from each sample was loaded onto 10% SDS-polyacrylamide gels and transferred to a nitrocellulose membrane (Schleicher and Schuell, Dassel, Germany). The membranes were incubated with the appropriate primary antibodies, and processed as described [27]. Densitometric analysis was performed using the Aida 2.1 Image® software.

#### Flow Cytometry Analysis

Mononuclear cell population was isolated from dissected muscles by enzymatic digestion. 10^6^ cells were incubated on ice with 1 µg of the anti- Mac-3 FITC-labelled and of the anti-CD45 PE-labelled antibodies (BD Biosciences, CA, USA) and analysed with a FacsStar Plus cytofluorimeter. Non-specific fluorescent emission was determined with the specific labelled isotypes.

### Treadmill exercise performance

Mice were first acclimated to the treadmill (LE 8710, PanLab S.L.U., Barcelona, Spain) before running by placing them on an unmoving treadmill for 10 min. The treadmill was set at a speed of 15 cm/sec. The test ran for 30 min, and the number of times at which mice failed to keep running, was recorded. Each test was performed twice a week for three weeks, and the results were averaged for each mouse.

### Bone Marrow transplantation

1 mo old *mdx/θ^−/−^* mice were X-irradiated (8 Gy per mouse) by a Siemens linear accelerator operating at 10 MV, at a rate of 3 Gy/min. At 2 h postirradiation, the mice were intravenously injected with bone marrow cell suspension derived from age-matching *mdx* or *mdx/θ^−/−^* mice. Mice were sacrificed 6 weeks after transplantation, and bone marrow, thymus and spleen were evaluated for exogenous cells accumulation. Different muscles were dissected for morphological and molecular analyses.

### PCR genotyping

Genomic DNA PCR, for PKCθ^−/−^ mutation, was conducted as previously described [36]. A 700 bp PCR fragment is expected for the mutated PKCθ gene, while a 400 bp PCR fragment for the WT one.

### Statistical analysis

Quantitative data are presented as means ± SEM or ± SD (as specified) of at least three independent experiments. Statistical analysis to determine significance was performed using paired Student's t tests. Differences were considered to be statistically significant at the *p<0.05* level.
